# Can physicochemical properties of antimicrobials be used to predict their pharmacokinetics during extracorporeal membrane oxygenation? Illustrative data from ovine models

**DOI:** 10.1186/s13054-015-1151-y

**Published:** 2015-12-15

**Authors:** Kiran Shekar, Jason A. Roberts, Adrian G. Barnett, Sara Diab, Steven C. Wallis, Yoke L. Fung, John F. Fraser

**Affiliations:** Critical Care Research Group, The Prince Charles Hospital, Rode Road, Chermside, QLD 4032 Australia; Discipline of Anaesthesiology and Critical Care, The University of Queensland, Brisbane, QLD Australia; Burns Trauma and Critical Care Research Centre, The University of Queensland, Brisbane, QLD Australia; Institute of Health and Biomedical Innovation, School of Public Health & Social Work, Queensland University of Technology, Brisbane, QLD Australia; School of Health and Sports Science, University of the Sunshine Coast, Sippy Downs, QLD Australia

## Abstract

**Introduction:**

Ex vivo experiments in extracorporeal membrane oxygenation (ECMO) circuits have identified octanol-water partition coefficient (logP, a marker of lipophilicity) and protein binding (PB) as key drug factors affecting pharmacokinetics (PK) during ECMO. Using ovine models, in this study we investigated whether these drug properties can be used to predict PK alterations of antimicrobial drugs during ECMO.

**Methods:**

Single-dose PK sampling was performed in healthy sheep (HS, *n* = 7), healthy sheep on ECMO (E24H, *n* = 7) and sheep with smoke inhalation acute lung injury on ECMO (SE24H, *n* = 6). The sheep received eight study antimicrobials (ceftriaxone, gentamicin, meropenem, vancomycin, doripenem, ciprofloxacin, fluconazole, caspofungin) that exhibit varying degrees of logP and PB. Plasma drug concentrations were determined using validated chromatographic techniques. PK data obtained from a non-compartmental analysis were used in a linear regression model to predict PK parameters based on logP and PB.

**Results:**

We found statistically significant differences in pH, haemodynamics, fluid balance and plasma proteins between the E24H and SE24H groups (*p* < 0.001). logP had a strong positive linear relationship with steady-state volume of distribution (V_ss_) in both the E24H and SE24H groups (*p* < 0.001) but not in the HS group (*p* = 0.9) and no relationship with clearance (CL) in all study groups. Although we observed an increase in CL for highly PB drugs in ECMO sheep, PB exhibited a weaker negative linear relationship with both CL (HS, *p* = 0.01; E24H, *p* < 0.001; SE24H, *p* < 0.001) and V_ss_ (HS, *p* = 0.01; E24H, *p* = 0.004; SE24H, *p* =0.05) in the final model.

**Conclusions:**

Lipophilic antimicrobials are likely to have an increased V_ss_ and decreased CL during ECMO. Protein-bound antimicrobial agents are likely to have reductions both in CL and V_ss_ during ECMO. The strong relationship between lipophilicity and V_ss_ seen in both the E24H and SE24H groups indicates circuit sequestration of lipophilic drugs. These findings highlight the importance of drug factors in predicting antimicrobial drug PK during ECMO and should be a consideration when performing and interpreting population PK studies.

## Introduction

With refinements in technology, extracorporeal membrane oxygenation (ECMO) and extracorporeal life support (ECLS) in general now represent a significant development in intensive care practice [1–4]. A wide range of acutely ill patients with cardiorespiratory failure are now being successfully rescued with ECLS therapies, but clinicians await definitive evidence supporting their use. Invasive ECLS therapies such as ECMO are complex supportive interventions, and outcomes rely not only on technology but also on user experience [5]; optimisation of other aspects of intensive care unit (ICU) management, established processes and other available services in each centre; and optimisation of pharmacotherapy to minimise and/or treat complications [6].

A variety of infectious and non-infectious conditions may result in severe cardiorespiratory failure, and an infection or sepsis is no longer considered a contraindication for ECMO [7]. Similarly, patients on ECMO may develop a variety of ICU-acquired infections that may necessitate antimicrobial therapy. Optimal antimicrobial therapy in these patients is a balance between potency, bacterial susceptibility and exposure [8, 9]. The authors of a recent review identified 30.1 infections per 1000 days of ECMO among patients with infections who were experiencing prolonged ICU and hospital lengths of stay [10]. The authors of another review [11] identified a total of 2418 infections in 20,741 (12 %) ECMO cases, with increased morbidity seen in patients with infections. Antimicrobial therapy is commonly prescribed in ECMO patients, and optimisation of dosing is central not only to improving patient outcomes but also to minimising the emergence of microbial resistance [8].

However, ECMO is known to induce significant pharmacokinetic (PK) alterations [12] in critically ill patients who already exhibit significantly altered PK [13], raising concerns of therapeutic failure or toxicity. Neonatal studies have shown major variations in antibiotic PK during ECMO [12, 14–16], and there is an emerging body of literature to support this in adult patients [17–20]. The interaction between the drug, the ECMO device and the disease are complex; hence clinical population PK studies alone may not be able to advance understanding of mechanisms behind altered PK in ECMO patients. This calls for systematic investigation [21] of each of these factors. To this end, experimental studies [22, 23] using circuit components used in adults have shown significant drug sequestration in ECMO circuits based on physicochemical properties of the drug, such as drug stability, octanol-water coefficient (logP, a marker of lipophilicity) and protein binding (PB).

Ex vivo experimental conditions are quite different from in vivo scenarios. The addition of an extracorporeal circuit to a critically ill patient may result in profound PK alterations, and appreciating the relative contributions of drug, device and disease factors to altered PK is challenging. Building on the data derived from ex vivo circuit studies, we aimed to develop PK models for antibiotic study drugs that exhibit wide a range of logP and PB in ambulatory healthy sheep (HS) as well as in healthy and critically ill sheep on ECMO. We hypothesised that the drug properties logP and PB can be used to predict PK alterations of antimicrobial drugs during ECMO.

## Methods

Ethical approval was obtained from the Queensland University of Technology Animal Ethics Committee (approval number 1100000053) and the University of Queensland Animal Ethics Committee (approval number QUT/194/12). All experimentation was done in accordance with the National Health and Medical Research Council’s Australian Code for the Care and Use of Animals for Scientific Purposes, Eighth Edition (2013) (https://www.nhmrc.gov.au/book/australian-code-care-and-use-animals-scientific-purposes-8th-edition-2013).

### Pharmacokinetic sampling

#### Healthy ambulatory sheep

Seven HS weighing 46–51 kg were housed in a metabolic cart amongst a larger flock, with free access to food and water. Two three-lumen central venous catheters were inserted in the left and right internal jugular veins (IJVs) while the animals were under local anaesthesia for drug administration and PK sampling. The catheters were secured with adhesive glue and a sleeve dressing around the neck. Study drugs were infused for 30 minutes, and serial blood samples were obtained for drug assays using validated chromatographic methods and subsequent PK analysis.

#### Healthy sheep on ECMO

We performed PK sampling in seven healthy sheep on extracorporeal membrane oxygenation (E24H). A detailed description of our ovine model of venovenous ECMO is provided elsewhere [21, 24]. Briefly, a central venous line was placed in the right IJV while the animals were under local anaesthesia. Alfaxalone, ketamine and midazolam were used for induction and maintenance of anaesthesia. Buprenorphine 0.01 mg/kg was used for supplemental analgesia. Sheep were intubated and ventilated with a Hamilton Galileo ventilator (Hamilton Medical AG, Bonaduz, Switzerland). The facial artery was cannulated for invasive arterial blood pressure monitoring. A pulmonary arterial catheter provided continuous measurements of central venous pressure, mixed venous oxygen saturation and continuous cardiac output (CCO).

Cannulation for ECMO was performed with the animals in supine position. A 21-French (50 cm) CARMEDA BioActive Surface–coated (CBAS®; Carmeda, Upplands Väsby, Sweden) venous cannula (Medtronic, Minneapolis, MN, USA) was inserted into the right IJV using a Seldinger technique and positioned using intra-cardiac echocardiography (ICE) [25] in the proximal inferior vena cava. A 19-French (50 cm) CARMEDA-coated femoral venous cannula was used for return blood and was inserted in the right IJV and positioned at the superior vena cava right atrium using ICE. ECMO pump speeds were titrated to target flows at least two-thirds of pre-ECMO CCO (or 60–80 ml/kg). Immediately upon commencement of ECMO, study drugs were infused for 30 minutes and serial blood samples were obtained for drug assays using validated chromatographic methods and subsequent PK analysis.

#### Smoke inhalation acute lung injury sheep on ECMO

We performed PK sampling in six sheep with smoke inhalation acute lung injury on ECMO (SE24H). The anaesthesia and ECMO techniques we used are described in the previous section. Smoke inhalation acute lung injury (S-ALI) was induced using a validated, reproducible technique previously published [26]. Briefly, a stainless steel plate was heated to 750 °C and placed on top of 8 g of cotton in a cup. The smoke resulting from combustion collected in the bellows of the purpose-built device was delivered to the sheep by manual compression (tidal volume [V_T_], 10–12 ml/kg) to achieve a carboxyhaemoglobin content of 45–50 %. The sheep were ventilated using Acute Respiratory Distress Syndrome Network criteria (V_T_ 4–6 ml/kg, positive end-expiratory pressure 10–15 cm H_2_O) for lung-protective ventilation [27]. Once ECMO was established, study drugs were infused for 30 minutes and serial blood samples were obtained for drug assays using validated chromatographic methods and subsequent PK analysis.

### Study drugs, drug administration and pharmacokinetic sampling

Following baseline sampling, study drugs in identical doses were administered to the HS, E24H and SE24H groups. The chosen anti-infective study drugs exhibit a wide range of logP and PB (Table 1). The intravenous (IV) study drugs (doses, administration techniques) used were meropenem (500 mg, bolus), ceftriaxone (500 mg, IV bolus), gentamicin (240 mg, slow IV bolus), vancomycin (500 mg in 50 ml 0.9 % saline, IV for 30 minutes), fluconazole (100 mg in 50 ml of 0.9 % saline, IV for 30 minutes), caspofungin (50 mg in 100 ml of 0.9 % saline, IV for 30 minutes), ciprofloxacin (100 mg in 50 ml of 0.9 % saline, IV for 30 minutes) and doripenem (500 mg in 100 ml of 0.9 % saline, IV for 30 minutes). Serial blood samples (2 ml) were obtained at 15, 30, 45, 60, 90, 180, 360, 480 and 720 minutes after commencement of antibiotic drug infusions for drug assays and subsequent PK analysis.

**Table 1:** Lipophilicity and protein binding characteristics of study drugs

Study drug	Lipophilicity (logP)	Protein binding (%)
Ceftriaxone	−1.7	95
Ciprofloxacin	2.3	20–40
Caspofungin	0.1	97
Fluconazole	0.4	11–12
Gentamicin	−3.1	0–30
Meropenem	−0.6	2
Doripenem	0.7	8
Vancomycin	−3.1	55

A higher numeric value for octanol-water partition coefficient (logP) indicates greater lipophilicity [29]

### Antimicrobial drug assays

Meropenem, doripenem, ceftriaxone and vancomycin analysis was done using high-performance liquid chromatography (HPLC) on a Prominence Ultra Fast system (Shimadzu, Kyoto, Japan) with ultraviolet light detection at 304 nm (meropenem and doripenem) and 230 nm (ceftriaxone and vancomycin). Ciprofloxacin was analysed on a Prominence HPLC system with fluorescence detection at 278 nm (excitation) and 456 nm (emission). Caspofungin, gentamicin and fluconazole analysis was carried out using liquid chromatography–tandem mass spectrometry on a Shimadzu Nexera-8030+ system with detection by positive mode multiple reaction monitoring. Samples were prepared by protein precipitation with trichloroacetic acid (ciprofloxacin and gentamicin), acetonitrile (caspofungin and fluconazole) or acetonitrile with dichloromethane washing (meropenem, ceftriaxone, vancomycin and doripenem). Chromatography was carried out using reversed-phase C18 HPLC columns (meropenem, ceftriaxone, vancomycin, doripenem, ciprofloxacin), reversed-phase C8 HPLC columns (caspofungin, fluconazole) or high-performance liquid chromatography (HPLC) (gentamicin). All methods were validated according to the guidelines of the US Food and Drug Administration [28]. All samples were assayed with internal standards, alongside calibration standards and quality control samples, and met the acceptance criteria.

### Statistical analysis and pharmacokinetic modelling

Discrete variables were expressed as count (percentage) and continuous variables as mean ± SD. Demographics and clinical differences between study groups were assessed using a χ^2^ test, Fisher’s exact test or Student’s *t* test, as appropriate. *p* < 0.05 was considered statistically significant.

A linear mixed effects model was used to examine changes in concentration over time whilst controlling for repeated results from the same sheep. The result adjusts for changes over time and repeated results from the same sheep. The concentration versus time curves (mean ± SEM) were plotted using GraphPad Prism version 5.03 software (GraphPad Software, La Jolla, CA, USA). PK analysis of antibiotic concentrations was undertaken using a non-compartmental approach. All statistical analyses were done using R version 3.1.2 software (R Foundation for Statistical Computing, Vienna, Austria).

We compared the statistical data between the three groups using a box plot. To look for a difference in the mean statistics between groups, we used a linear model with group as the dependent variable. Because the PK data were strongly positively skewed, we log-transformed them before building regression models. Regression models were derived to examine the differences in the following PK parameters between the three study groups: area under the curve (AUC), mean resident time, clearance (CL), steady-state volume of distribution (V_ss_), maximum plasma concentration and minimum plasma concentration. A linear regression analysis was used to predict PK parameters based on drug properties. logP data for the individual drugs are available from the University of Alberta DrugBank website [29].

## Results

We observed no complications during the ECMO run. We found no significant differences between the physiologic variables at the baseline (Table 2). Differences in physiologic variables between the E24H and SE24H groups are presented in Table 3. We found statistically significant differences in pH, haemodynamics, fluid balance and plasma proteins between the E24H and SE24H groups (*p* < 0.001).

**Table 2:** Demographic and physiologic data at baseline after initiation of anaesthesia, mechanical ventilation and haemodynamic monitoring and before smoke inhalation and commencement of ECMO

	Group	Mean	SD	*p* Value
Weight, kg	E24H	48.5	4.6	0.84
	SE24H	49.6	4.4	
Heart rate, beats/min	E24H	116	13	0*.*78
	SE24H	118	11	
Mean arterial BP, mmHg	E24H	116*.*1	6*.*8	0*.*91
	SE24H	115*.*7	8*.*1	
Mean PAP, mmHg	E24H	24*.*6	2*.*8	0*.*06
	SE24H	21*.*2	3*.*2	
CVP, cmH_2_O	E24H	15*.*6	2*.*8	0*.*06
	SE24H	12*.*2	3*.*2	
CCO, L/min	E24H	5*.*44	0*.*93	0*.*33
	SE24H	5*.*03	0*.*46	
SvO_2_, %	E24H	78*.*8	7*.*1	0*.*31
	SE24H	82*.*0	4*.*1	
PEEP, cmH_2_O	E24H	8*.*1	2*.*6	0*.*67
	SE24H	7*.*5	2*.*7	
Respiratory rate, breaths/min	E24H	10*.*9	6*.*0	0*.*6
	SE24H	12*.*5	5*.*2	
Fluid balance, ml	E24H	681	376	0*.*11
	SE24H	926	68	
Haemoglobin, g/L	E24H	7*.*0	1*.*2	0*.*47
	SE24H	7*.*6	1*.*7	
pH	E24H	7*.*385	0*.*031	0*.*62
	SE24H	7*.*397	0*.*052	
Body temperature, °C	E24H	38*.*26	0*.*61	0*.*67
	SE24H	38*.*13	0*.*50	
Lactate, mmol/L	E24H	1*.*34	0*.*44	0*.*17
	SE24H	1*.*07	0*.*24	
Midazolam dose, mg/h	E24H	14*.*4	1*.*2	0*.*17
	SE24H	15*.*0	0*.*0	
Urine output, ml/h	E24H	69	44	0*.*87
	SE24H	74	68	
Albumin, g/L	E24H	37*.*16	2*.*53	0*.*33
	SE24H	38*.*15	0*.*86	
Alanine aminotransferase (U/L)	E24H	11*.*7	2*.*1	0*.*51
	SE24H	12*.*4	1*.*7	
Serum bilirubin, μmol/L	E24H	2*.*16	0*.*69	0*.*75
	SE24H	2*.*03	0*.*74	
Serum creatinine, μmol/L	E24H	88	15	0*.*94
	SE24H	87	11	
Total protein, g/L	E24H	71*.*7	6*.*4	0*.*78
	SE24H	71*.*0	1*.*9	
Urine creatinine, μmol/L	E24H	10,172	3798	0*.*17
	SE24H	15,541	8009	

*BP* blood pressure, *PAP* pulmonary arterial pressure, *CVP* central venous pressure, *CCO* continuous cardiac output, SvO_2_ mixed venous oxygen saturation, *PEEP* positive end-expiratory pressure

Data presented are derived from comparison of the results between groups: healthy sheep on extracorporeal membrane oxygenation (E24H) (*n* = 7), sheep with smoke inhalation acute lung injury on extracorporeal membrane oxygenation (SE24H) (*n* = 6)

**Table 3:** Mean differences in Physiologic parameters of E24H and SE24H groups during the pharmacokinetic sampling interval

Variable	Mean	Lower	Upper	*p* Value
Heart rate	1.41	−9.54	12.36	0.815
Mean arterial BP	−23.83	−31.35	−16.25	*<*0.001
Mean PAP	1.77	−0.83	4.37	0.234
CVP	0.93	−3.00	4.86	0.669
CCO	−2.09	−3.30	−0.87	0.01
SvO_2_	−0.78	−3.31	1.74	0.585
PEEP	2.09	0.66	3.52	0.02
Respiratory rate (sheep)	2.33	0.18	4.48	0.068
Tidal volume	−38.24	−135.38	58.87	0.479
FiO_2_	7.77	−2.04	17.69	0.151
paO_2_	48.05	0.43	95.67	0.053
paCO_2_	3.12	−0.28	6.52	0.115
Running fluid balance	4604.14	2779.38	6428.89	*<*0.001
ctHb	2.00	1.34	2.66	*<*0.001
pH	−0.07	−0.10	−0.03	0.003
Body temperature	−0.06	−0.38	0.27	0.754
Lactate	0.71	0.26	1.16	0.013
Midazolam dose per hour	−5.97	−21.37	9.42	0.485
Urine output	6.68	−54.88	68.25	0.843
Albumin	−14.91	−16.70	−13.10	*<*0.001
ALT	5.12	−6.25	16.49	0.419
AST	82.87	−23.66	189.39	0.175
Bilirubin (Direct)	−0.48	−1.68	0.73	0.476
Bilirubin (Total)	−0.12	−0.71	0.50	0.823
Creatinine	0.56	−11.52	12.64	0.933
Total protein	−26.60			*<*0.001
Urea	0.79	−0.47	2.06	0.271
Urine creatinine	−4327.47	−9508.52	974.49	0.322

*BP* blood pressure, *PAP* pulmonary arterial pressure, *CVP* central venous pressure, *CCO* continuous cardiac output, *SvO*_*2*_ mixed venous oxygen saturation, *PEEP* positive end-expiratory pressure, *FiO*_*2*_ fraction of inspired oxygen, *paO*_*2*_ partial pressure of oxygen, *paCO*_*2*_ partial pressure of carbon dioxide, *ctHb* concentration of total blood haemoglobin, *ALT* alanine aminotransferase, *AST* aspartate aminotransferase

The analysis was carried out using a mixed model with a random intercept for each sample. The results are presented as mean difference and 95 % confidence intervals, including a linear time trend and using each sheep’s baseline (time 0) as a covariate

**Fig. 1:**
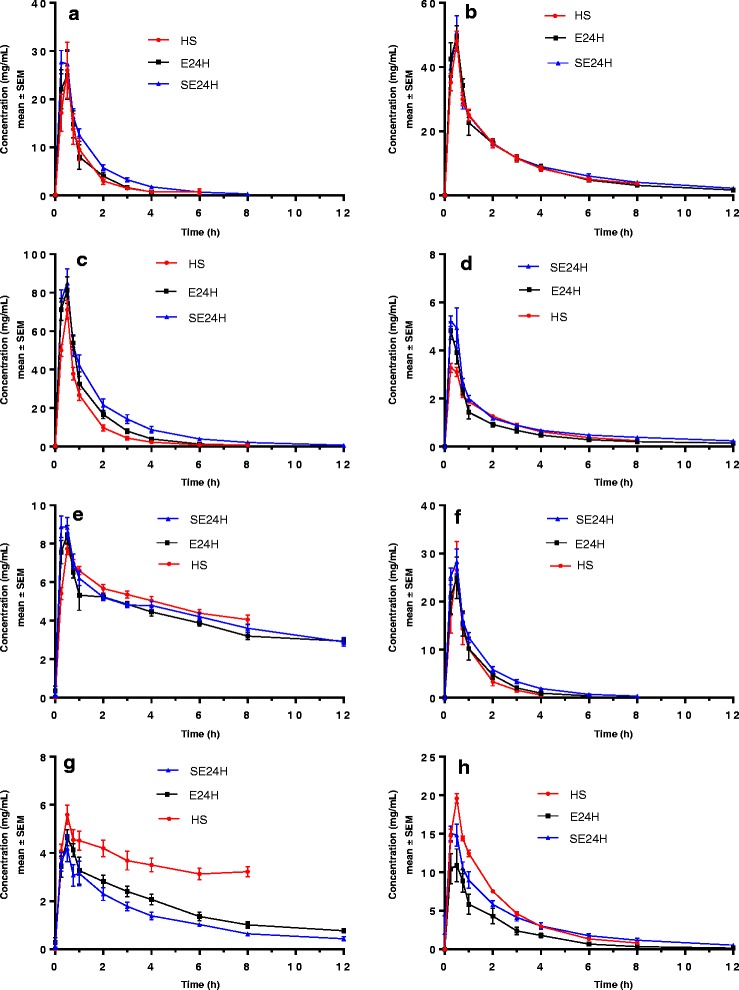
Concentration versus time curves for study drugs. **a** Meropenem. **b** Vancomycin. **c** Ceftriaxone. **d** Ciprofloxacin. **e** Fluconazole. **f** Doripenem. **g** Caspofungin. **h** Gentamicin. *E24H* healthy sheep on extracorporeal membrane oxygenation, *HS* healthy sheep, *SE24H* sheep with smoke inhalation acute lung injury on extracorporeal membrane oxygenation

Sixteen hundred samples were analysed for study drug concentrations. Concentration versus time curves for the study antibiotics are shown in Fig. 1. A summary of PK parameters estimated using a non-compartmental analysis is presented in Table 4. Significant differences in AUC between groups were found for ciprofloxacin, gentamicin and caspofungin. For ciprofloxacin, the most lipophilic drug studied, there was a significant difference in V_ss_ between the E24H and SE24H groups (*p* = 0.004). For relatively protein-bound drugs, there was a trend towards increased V_ss_ only in the SE24H group compared with HS group. However, an increase in CL was seen in both the E24H and SE24H groups compared with the HS group for vancomycin (*p* = 0.02 for both), ceftriaxone (*p* = 0008 and *p* = 0.05, respectively) and caspofungin (*p* < 0.001 for both), which are relatively more protein-bound.

**Table 4:** Non-compartmental pharmacokinetic estimates for eight study drugs for all three study groups

Study drug	Group	C_max_ (mg/L)	C_min_ (mg/L)	AUC_0–∞_ (mg/h/L)	V_ss_ (L)	Clearance (L/h)	MRT (h)
Ceftriaxone	HS	71 (14)	0.0 (0.1)	202 (46)	9.0 (1.8)	2.6 (0.6)	3.5 (0.2)
	E24H	86 (15)	1.2 (1.4)	135 (74)^a^	6.3 (1.4)^a^	4.3 (1.4)^a^	1.7 (0.8)^a^
	SE24H	85 (18)	1.0 (0.3)^b,c^	142 (39)	7.4 (1.8)	3.6 (0.8)	2.1 (0.6)^b^
Vancomycin	HS	48 (9)	0.0 (0)	180 (37)	12.4 (2.6)	2.8 (0.7)	4.5 (0.2)
	E24H	52 (10)	1.59 (0.9)^a^	131 (60)^a^	15.7 (3.9)	4.0 (1.1)^a^	4.0 (0.6)
	SE24H	51 (12)	2.2 (0.4)^b^	116 (20)^b^	19.3 (3.3)^b^	3.9 (0.6)^b^	4.9 (0.7)^c^
Gentamicin	HS	20 (2)	0.0 (0)	78 (7)	13.0 (1.3)	3.1 (0.3)	4.3 (0.1)
	E24H	12 (6)^a^	0.3 (0.3)^a^	25 (12)^a^	34 (23.5)^a^	12.1 (7.4)^a^	2.7 (0.5)^a^
	SE24H	16 (2)^b^	0.6 (0.2)^b,c^	39 (6.5)^b^	20.1 (2.9)^b^	5.9 (1.0)^b,c^	3.5 (0.7)^b,c^
Meropenem	HS	26 (16)	0.0 (0.1)	71 (45)	91.0 (138)	20.9 (28)	3.9 (0.8)
	E24H	27 (13)	0.5 (0.4)	36 (26)	16.5 (3.0)	13.8 (4.9)	1.4 (0.9)^a^
	SE24H	29 (7)	0.3 (0.1)	39 (9.0)	19.9 (3.7)	13.2 (2.4)	1.6 (0.3)^b^
Doripenem	HS	26 (17)	0 (0)	73 (47)	63.8 (83)	17.6 (22)	3.5 (0.2)
	E24H	30 (6)	0 (0)	42 (20)	17.1 (2.8)	13.5 (4.6)	1.5 (0.9)^a^
	SE24H	28 (6)	0 (0)	39 (8)	20.2 (3.0)	13.1 (2.4)	1.6 (0.2)^b^
Ciprofloxacin	HS	3.3 (0.5)	0 (0)	13.6 (1.8)	31.9 (4.4)	7.2 (0.9)	4.5 (0.3)
	E24H	5.1 (1.1)^a^	0.1 (0.1)^a^	8.3 (1.5)^a^	39.0 (7.6)^a^	11.8 (2.5)^a^	3.5 (1.2)^a^
	SE24H	5.8 (1.2)^b^	0.1 (0.1)^b,c^	10.2 (1.5)^b,c^	52.7 (9.1)^b,c^	8.2 (1.2)^c^	6.4 (0.5)^b,c^
Fluconazole	HS	7.7 (0.9)	0 (0)	48.2 (6.2)	13.3 (2.2)	1.2 (0.3)	12.0 (5.8)
	E24H	9.1 (1.2)^a^	2.6 (0.8)^d^	51.0 (5.0)	16.7 (2.8)^a^	1.0 (0.3)	17.1 (5.5)
	SE24H	9.2 (1.4)^b^	2.9 (0.5)^b^	52.3 (3.5)	17.7 (3.4)^b^	0.8 (0.5)^b^	33.7 (29)^b^
Caspofungin	HS	5.7 (1.0)	0 (0)	33.8 (7.3)	10.0 (2.5)	0.8 (0.1)	12.9 (2.6)
	E24H	4.8 (0.8)	0.7 (0.2)^a^	22.3 (6.6)^a^	14.4 (5.0)^a^	1.9 (0.4)^a^	7.6 (1.9)^a^
	SE24H	4.3 (1.3)^b^	0.4 (0.2)^b,c^	15.5 (3.7)^b,c^	18.8 (8.4)^b^	2.8 (0.9)^b,c^	7.9 (6.4)^b^

*HS* healthy sheep (*n* = 7), *E24H* healthy sheep on extracorporeal membrane oxygenation (*n* = 7), *SE24H* sheep with smoke inhalation acute lung injury on extracorporeal membrane oxygenation (*n* = 6), *AUC* area under the curve, *MRT* mean resident time, *V*_*ss*_ steady-state volume of distribution, *C*_*max*_ maximum plasma concentration, *C*_*min*_ minimum plasma concentration

^a^Statistically significant results for E24H group compared with HS group

^b^Statistically significant results for SE24H group compared with HS group

^c^Statistically significant differences between E24H and SE24H groups

**Fig. 2:**
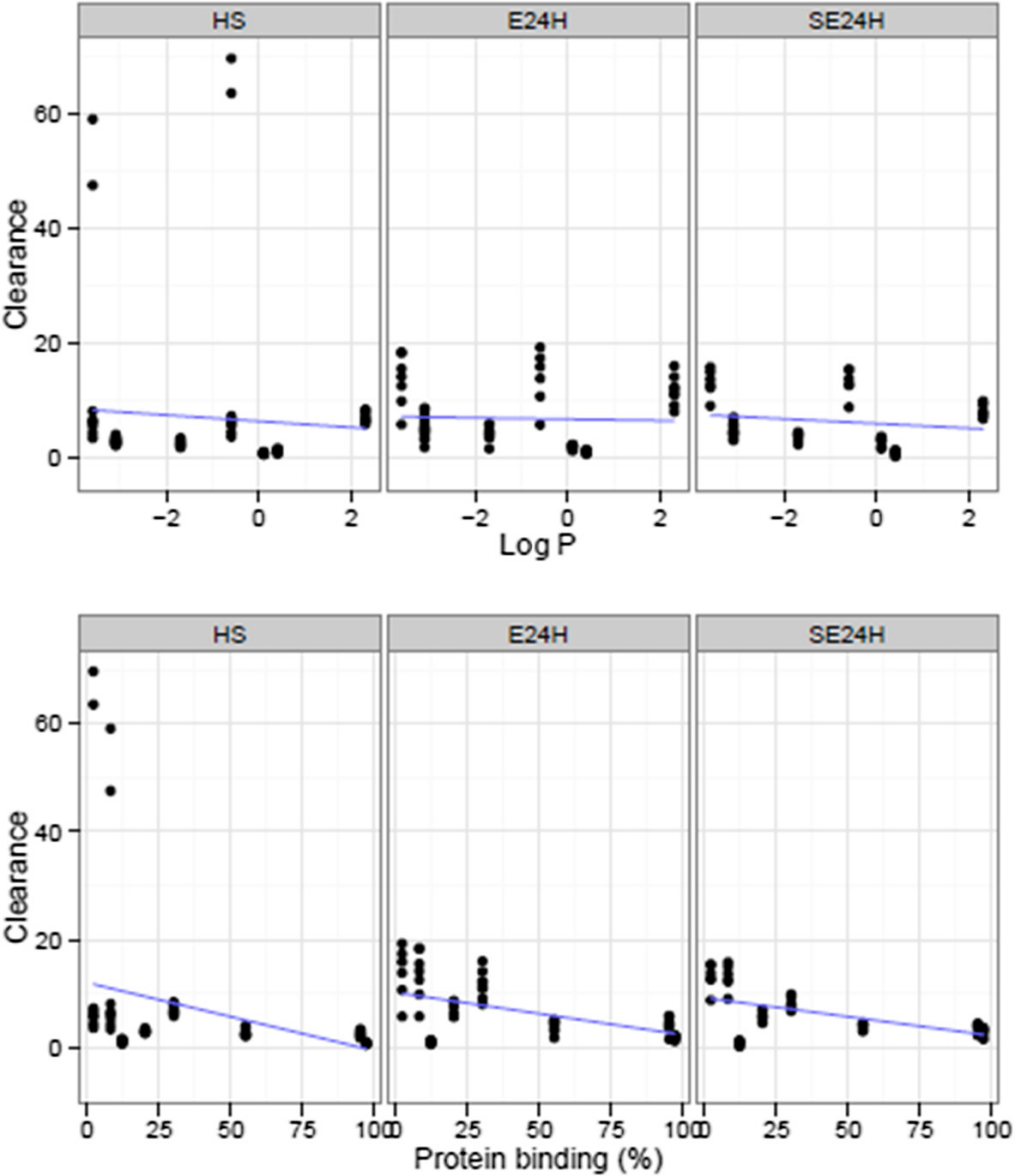
Scatterplots and regression of clearance against octanol-water partition coefficient and protein binding by groups. Healthy sheep (HS, *n* = 7), healthy sheep on extracorporeal membrane oxygenation (E24H, *n* = 7), sheep with smoke inhalation acute lung injury on extracorporeal membrane oxygenation (SE24H, *n* = 6)

**Fig. 3:**
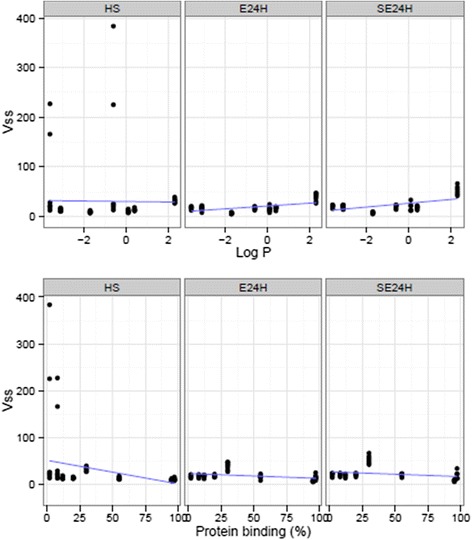
Scatterplots and regression of steady-state volume of distribution (V_ss_) against octanol-water partition coefficient and protein binding by groups. Healthy sheep (HS, *n* = 7), healthy sheep on extracorporeal membrane oxygenation (E24H, *n* = 7), sheep with smoke inhalation acute lung injury on extracorporeal membrane oxygenation (SE24H, *n* = 6)

Scatterplots and linear regression of both CL and V_ss_ against logP and PB are presented in Figs. 2 and 3. Table 5 shows regression parameters for predicting study drug PK using logP and PB. PB exhibited a weaker negative linear relationship with CL (HS, *p* = 0.01; E24H, *p* < 0.001; SE24H, *p* < 0.001) and with V_ss_ (HS, *p* = 0.01; E24H, *p* = 0.004; SE24H, *p* =0.05). Despite an increased CL for more protein-bound study drugs, PB in itself was a predictor of decreased CL in all study groups (Table 5). logP had a strong positive linear relationship with V_ss_ in both E24H and SE24H (*p* < 0.001) but not in HS (*p* = 0.9). There was no significant association of logP with CL (HS, *p* = 0.55; E24H, *p* = 0.74; SE24H, *p* = 0.24).

**Table 5:** Linear regression parameters for predicting PK parameters using drug properties

Group	Dependent	Independent	Mean	Lower	Upper	*p* Value
HS	CL	logP	−0.54	−2.37	1.29	0.556
E24H	CL	logP	−0.12	−0.86	0.62	0.744
SE24H	CL	logP	−0.41	−1.09	0.28	0.235
HS	CL	PB	−0.13	−0.22	−0.03	0.01
E24H	CL	PB	−0.08	−0.11	−0.04	*<*0.001
SE24H	CL	PB	−0.07	−0.10	−0.04	*<*0.001
HS	V_ss_	logP	−0.48	−8.43	7.46	0.903
E24H	V_ss_	logP	2.85	1.73	3.97	*<*0.001
SE24H	V_ss_	logP	3.84	2.18	5.50	*<*0.001
HS	V_ss_	PB	−0.50	−0.92	−0.09	0.017
E24H	V_ss_	PB	−0.10	−0.17	−0.04	0.004
SE24H	V_ss_	PB	−0.10	−0.21	0.00	0.056

*PB* protein binding, *logP* octanol-water partition coefficient (measure of drug lipophilicity)

Separate results for each group are presented for healthy sheep (HS, *n* = 7), healthy sheep on extracorporeal membrane oxygenation (E24H, *n* = 7), sheep with smoke inhalation acute lung injury on extracorporeal membrane oxygenation (SE24H, *n* = 6) and pharmacokinetic (PK) parameters clearance (CL) and steady-state volume of distribution (V_ss_)

## Discussion

In this study, we systematically investigated the effects of the ECMO circuit on PK in HS and the combined effects of ECMO circuit and critical illness on PK in S-ALI sheep receiving ECMO. In addition, by using antimicrobials with a range of logP and PB, we were also able to investigate the relative contributions of drug, circuit and disease factors influencing PK during ECMO.

There was some expected variability in PK parameters between the groups. Overall, the main findings of the study are that (1) a significant increase in V_ss_ for lipophilic drugs that was observed only in the ECMO sheep and (2) protein-bound drugs exhibited decreased CL and CL was also more significantly reduced in ECMO sheep. These findings are significant, as they conform to PK alterations described in neonates in the clinical ECMO setting and to emerging PK data in adults, and they provide further insights into mechanisms behind these PK alterations.

Although an increase in V_ss_ during ECMO has been described clinically [12] for many antimicrobial and sedative drugs, the relative contribution of critical illness, circuit and drug factors towards this phenomenon is largely unclear. Systemic inflammation, capillary leak syndrome and hypoproteinaemia during critical illness can result in a significantly increased V_ss_ [13]. Similarly, sequestration of drugs in ECMO circuits may lead to a further increase in V_ss_. Equally, a reduction in drug CL during critical illness may result from renal and hepatic dysfunction [13]. This study confirms both these findings. An increase in V_ss_ for lipophilic drugs occurred in both E24H and SE24H but not in HS, clearly highlighting the role of circuit drug sequestration. For all study drugs except ciprofloxacin, we found no significant difference in V_ss_ between the E24H and SE24H groups, hence the additional influence of critical illness, if at all, in increasing V_ss_ was less apparent. The reasons behind a greater V_ss_ seen in the case of ciprofloxacin in the SE24H group relative to the E24H group is probably a result of decreased CL in the SE24H group and may indicate altered hepatic metabolism. It should be noted that there was no biochemical evidence of any significant hepatic dysfunction in our model. Clinicians should consider circuit sequestration and alterations in hepatic function when prescribing lipophilic antibiotics. In patients with presumably preserved hepatic function, lipophilic antibiotics may have to be prescribed in higher doses. These findings need further validation in clinical PK studies.

Even though protein-bound drugs have previously been shown to be sequestered in ECMO circuits under physiologic conditions [23] with expected increased V_ss_, we observed no significant increase in V_ss_ for these drugs in the present study. However, there was a trend towards increased V_ss_ for protein-bound drugs in the SE24H group. This may have resulted from reduced plasma protein concentrations in the SE24H group. The difference in blood pH between the SE24H and E24H groups was significant and may have affected PB [30, 31] and circuit sequestration. Given that unbound drug concentrations were not measured, further interpretation of these data is not possible. From a general PK perspective, protein-bound drugs are expected to have a relatively lower V_ss_, and during critical illness and ECMO there is a potential for this to increase due to circuit sequestration and other critical illness–induced PK alterations [13]. The net increase in V_ss_ in a critically ill patient on ECMO is therefore challenging to predict on the basis of mechanistic studies alone. Clinical population PK studies are therefore indicated.

Decreases in CL of antimicrobial and other drugs during ECMO have been reported in previous clinical studies [12]. Antimicrobial CL could not be predicted on the basis of logP in the present study, which suggests that the CL for lipophilic drugs may depend largely on critical illness factors and hepatic drug metabolism. Sequestration of drugs in the ECMO circuit by itself is unlikely to play any significant role in reducing CL for lipophilic drugs. However, it should be noted that alterations in hepatic blood flow [32] and hepatic dysfunction may occur in patients before initiation of ECMO or during ECMO (especially during venoarterial ECMO initiated in patients with severe cardiac failure), which may then adversely affect hepatic metabolism of lipophilic drugs and result in decreased CL. The degree of biochemical hepatic derangement in the SE24H group that received venovenous ECMO for predominant respiratory failure may not have been sufficient to influence metabolism of lipophilic drugs significantly.

Even though protein-bound drugs appeared to have more significantly reduced CL in ECMO sheep in the final model, we observed increased CL in both healthy and critically ill sheep on ECMO for relatively more protein-bound drugs (55 % for vancomycin, 95 % for ceftriaxone and 97 % for caspofungin) compared with HS. Interestingly, these three drugs also demonstrated a trend towards an increased V_ss_ during ECMO, especially in the SE24H group. This is an interesting finding, given that protein-bound drugs have been shown to have a greater propensity for sequestration in ECMO circuits in the ex vivo setting. This relative increase in CL and a trend towards an increased V_ss_ for more protein-bound drugs in ECMO sheep may indicate circuit sequestration. Equally, an increase in plasma unbound fraction of these drugs due to heparin displacement [33] may also have contributed to increased CL and V_ss_ for these drugs. Although this increased CL was apparent in our ovine ECMO models with relatively preserved renal function, this may be of less significance in critically ill patients with significant renal dysfunction or those on continuous renal replacement therapy (CRRT). For example, no significant impact of ECMO on vancomycin CL was observed in a recent clinical population PK study by Donadello et al. [18]. The sheep had normal renal function, at least biochemically, as opposed to 7 of 11 patients who received CRRT in the above-mentioned study. This is an important point to note because kidney injury and relatively lower CL for vancomycin achieved on CRRT may have negated an increase in CL during ECMO.

Although ex vivo studies confirm relative stability of vancomycin in ECMO circuits, they do not replicate the in vivo situation. A recent ex vivo study showed that, with drugs with similar PB, lipophilicity becomes the determinant of eventual circuit loss. Vancomycin, although relatively protein-bound (55 %), is hydrophilic. Hence, it is possible that, in the in vivo setting, there is a greater propensity for hydrophilic protein-bound drugs to undergo circuit sequestration. Appropriately powered clinical population PK studies in which investigators compare vancomycin PK in ECMO patients with and without preserved renal function are needed to address this further, and such studies are currently underway [34].

In summary, sequestration of lipophilic antibiotics plays an important role in increasing their V_ss_ during ECMO. CL of lipophilic drugs is largely dependent on hepatic drug metabolism, which can be significantly affected in a subgroup of ECMO patients receiving venoarterial ECMO for cardiac failure. Although more protein-bound drugs were found to have relatively higher CL in this study, PB in isolation may not be a reliable predictor of CL. Patients on ECMO may have significant renal dysfunction, which is more likely to influence the net CL than sequestration alone. Overall, ECMO appears to decrease antimicrobial CL. These findings need further validation in clinical studies, and such studies are currently underway [34].

This animal study has limitations. Apart from inherent PK variability that is expected in a small sample, the distribution, metabolism and excretion processes in sheep may differ from those of humans. Despite the SE24H group’s development of severe cardiorespiratory failure following S-ALI, the degree of hepatic and renal dysfunction may not have been sufficient to more fully elucidate the full impact of critical illness of PK. However, the changes in PK due to critical illness are very well described, and the use of a model with no advanced end-organ failures that is designed to more fully examine the circuit–drug interactions is justified. Also, this study was directed more at observing relative PK changes between groups and the effects of drug factors logP and PB on antibiotic PK.

## Conclusions

Lipophilic antimicrobial agents are likely to have an increased V_ss_ and decreased CL during ECMO. Protein-bound antibiotics are likely to have reductions in both CL and V_ss_ during ECMO. The strong relationship between logP and V_ss_ during ECMO indicates circuit sequestration of lipophilic drugs. These findings highlight the importance of drug factors in predicting antibiotic drug PK during ECMO and should be a consideration when performing and interpreting population PK studies.

## Key messages

- Sequestration of lipophilic antibiotics results in increased V_ss_ on ECMO.
- Lipophilic drugs exhibit a larger V_ss_ during ECMO, and lipophilicity by itself has little impact on drug CL.
- Protein-bound drugs may have decreased V_ss_ and CL during ECMO.
- Higher doses of lipophilic antibiotics may be indicated in patients with intact hepatic function.
- Lipophilicity and PB are useful drug factors to use in predicting antibiotic PK during ECMO.

## Abbreviations

ALT: alanine aminotransferase
AST: aspartate aminotransferase
AUC: area under the curve
BP: blood pressure
CL: clearance
C_max_: maximum plasma concentration
C_min_: minimum plasma concentration
CCO: continuous cardiac output
CRRT: continuous renal replacement therapy
ctHb: concentration of total blood haemoglobin
CVP: central venous pressure
E24H: healthy sheep on extracorporeal membrane oxygenation
ECLS: extracorporeal life support
ECMO: extracorporeal membrane oxygenation
FiO_2_: fraction of inspired oxygen
HPLC: high-performance liquid chromatography
HS: healthy sheep
ICE: intra-cardiac echocardiography
ICU: intensive care unit
IJV: internal jugular vein
IV: intravenous
logP: octanol-water partition coefficient
MRT: mean resident time
paCO_2_: partial pressure of carbon dioxide
paO_2_: partial pressure of oxygen
PAP: pulmonary arterial pressure
PB: protein binding
PEEP: positive end-expiratory pressure
PK: pharmacokinetic(s)
S-ALI: smoke inhalation acute lung injury
SE24H: sheep with smoke inhalation acute lung injury on extracorporeal membrane oxygenation
SvO_2_: mixed venous oxygen saturation
V_ss_: steady-state volume of distribution
V_T_: tidal volume
